# Clinical Course and Management of Patients with Emergency Surgery Treated with Direct Oral Anticoagulants or Vitamin K Antagonists—Results of the German Prospective RADOA-Registry

**DOI:** 10.3390/jcm13010272

**Published:** 2024-01-03

**Authors:** Jana Last, Eva Herrmann, Ingvild Birschmann, Simone Lindau, Stavros Konstantinides, Oliver Grottke, Ulrike Nowak-Göttl, Barbara Zydek, Christian von Heymann, Ariane Sümnig, Jan Beyer-Westendorf, Sebastian Schellong, Patrick Meybohm, Andreas Greinacher, Edelgard Lindhoff-Last

**Affiliations:** 1 Coagulation Research Centre Bethanien Hospital, 63089 Frankfurt, Germany; b.zydek@ccb.de (B.Z.); e.lindhoff-last@ccb.de (E.L.-L.); 2 Deutsches Herzzentrum der Charité (DHZC) Berlin, Department of Cardiology, Angiology and Intensive Care Medicine, Charité Berlin, 12203 Berlin, Germany; 3 Institute of Biostatistics and Mathematical Modelling, Goethe University Frankfurt, 60596 Frankfurt, Germany; herrmann@med.uni-frankfurt.de; 4 Institute for Laboratory and Transfusion Medicine, Heart and Diabetes Centre, Ruhr University, 44801 Bochum, Germany; birschmann@hdz-nrw.de; 5 Department of Anaesthesiology, Intensive Care Medicine and Pain Therapy, University Hospital Frankfurt, 60596 Frankfurt, Germany; simone.lindau@kgu.de; 6 Center for Thrombosis and Hemostasis (CTH), University Medical Center of the Johannes Gutenberg University Mainz, 55122 Mainz, Germany; stavros.konstantinides@unimedizin-mainz.de; 7 Department of Anaesthesiology, RWTH Aachen University Hospital, 52062 Aachen, Germany; ogrottke@ukaachen.de; 8 Institute of Clinical Chemistry, Thrombosis & Hemostasis Treatment Centre, University Hospital, Kiel-Lübeck, 24105 Kiel, Germany; ulrike.nowak-goettl@uksh.de; 9 Coagulation Centre at the Cardiology Angiology Centre Bethanien Hospital (CCB), 63089 Frankfurt, Germany; 10 Department of Anaesthesia, Intensive Care Medicine, Emergency Medicine and Pain Therapy, Vivantes Klinikum im Friedrichshain, 10249 Berlin, Germany; christian.heymann@vivantes.de; 11 Institute for Transfusions Medicine, Universitätsmedizin Greifswald, 17489 Greifswald, Germany; ariane.suemnig@med.uni-greifswald.de (A.S.); andreas.greinacher@med.uni-greifswald.de (A.G.); 12 Department of Medicine 1, Division of Thrombosis & Hemostasis, Dresden University Clinic, 01307 Dresden, Germany; jan.beyer@uniklinikum-dresden.de; 13 Medical Department 2, Municipal Hospital, 01129 Dresden, Germany; sebastian.schellong@klinikum-dresden.de; 14 Department of Anaesthesiology, Intensive Care, Emergency and Pain Medicine, University Hospital Wuerzburg, 97080 Wuerzburg, Germany; meybohm_p@ukw.de

**Keywords:** emergency surgery, direct oral anticoagulants, vitamin K antagonists, major bleeding, prothrombin complex concentrate

## Abstract

(1) Background: The clinical management of anticoagulated patients treated with direct oral anticoagulants (DOAC) or Vitamin K antagonists (VKA) needing emergency surgery is challenging. (2) Methods: The prospective German RADOA registry investigated treatment strategies in DOAC- or VKA-treated patients needing emergency surgery within 24 h after admission. Effectiveness was analysed by clinical endpoints including major bleeding. Primary observation endpoint was in hospital mortality until 30 days after admission. (3) Results: A total of 78 patients were included (DOAC: 44; VKA: 34). Median age was 76 years. Overall, 43% of the DOAC patients and 79% of the VKA patients were treated with prothrombin complex concentrates (PCC) (*p* = 0.002). Out of the DOAC patients, 30% received no hemostatic treatment compared to 3% (1/34) of the VKA patients (*p* = 0.002), and 7% of the DOAC patients and 21% of the VKA patients developed major or clinically relevant non-major bleeding at the surgical site (*p* = 0.093). In-hospital mortality was 13% with no significant difference between the two treatment groups (DOAC: 11%, VKA: 15%; *p* > 0.20). (4) Conclusions: The 30-day in-hospital mortality rate was comparable between both patient groups. VKA patients required significantly more hemostatic agents than DOAC patients in the peri- and postoperative surgery period.

## 1. Introduction

Patients with nonvalvular atrial fibrillation, deep vein thrombosis or pulmonary embolism require anticoagulation with either vitamin K antagonists (VKA) or direct oral anticoagulants (DOACs) [1,2,3,4,5]. With the demographical change of a worldwide increase in aging patients, the prevalence of anticoagulated patients rises. In international guidelines, DOACs are recommended preferentially to VKA as first choice anticoagulant for these indications [6,7,8] but both VKA- and DOAC-treatments require dedicated hemostatic management in case of emergencies. Each year, about 0.5–1% of anticoagulated patients need emergency surgery [9]. However, clinical management of these patients is highly diverse.

Perioperative management may include factor concentrates such as prothrombin complex concentrates (PCC), fresh frozen plasma (FFP), transfusion of red blood cells (RBC) and/or platelet concentrates, and other hemostatic agents such as vitamin K or tranexamic acid. Furthermore, the reversal agent idarucizumab is now available for dabigatran, although it is rather expensive. [10,11]. Andexanet alfa, the specific reversal agent for direct oral FXa inhibitors is not approved in patients requiring emergency surgery [11].

Other strategies to enable surgery in anticoagulated patients include delaying the procedure, for which either laboratory tests (anticoagulation intensity or drug plasma concentrations) or pharmacokinetic (pk) considerations are taken into account [12,13]. However, delaying surgery may be associated with increased morbidity and mortality [14], e.g., after hip fracture.

Here, we provide data from the prospective German RADOA-registry (Reversal Agent use in patients treated with Direct Oral Anticoagulants or vitamin K antagonists Registry) on clinical practice, effectiveness, and safety of different treatment strategies in VKA- or DOAC-treated patients who needed emergency surgery.

## 2. Materials and Methods

The prospective RADOA-registry is a German observational, noninterventional, open-label, investigator-initiated, multicenter registry, which documents the management of severe bleeding and/or emergency surgery in patients treated with the vitamin K antagonist phenprocoumon or DOAC [15]. Here, we report data from the subgroup of patients who needed emergency surgery within 24 h after admission. Patients requiring surgery because of severe bleeding were excluded.

### 2.1. Patients

The inclusion criteria were:

- Age > 18 years.
- Patients anticoagulated with a DOAC or phenprocoumon needing an urgent surgical intervention (not caused by severe bleeding) within 24 h after admission.

### 2.2. Study Design and Oversight

Patients were followed prospectively up to 30 days after hospital admission. Participating centers were included only if they had 24 h interdisciplinary teams available to manage anticoagulant-related bleeding in specialized units (i.e., emergency departments and intensive care units). Patients were recruited at 10 German centers from April 2014 to March 2018. Clinical outcome measures were peri- and/or post-operative major bleeding, and requirement of hemostatic agents (PCC, FFP, tranexamic acid, etc.) or drug-specific antidotes.

The prespecified primary observation endpoint was 30-day in-hospital mortality (deaths that occurred within 30 days of hospitalization). Secondary observation endpoints were blood loss, number of blood transfusions, satisfaction of the surgeon with peri- and postoperative hemostasis, outcome after use versus no use of reversal agents, frequency of serious adverse events (SAE) depending on the type of anticoagulant, and delay of the procedures due to anticoagulation.

### 2.3. Definition of INR-Rebound

In patients treated with phenprocoumon, the INR was monitored according to the instructions of the treating physicians. An INR rebound was assumed if the INR value rose to >2.0 within 10 days after admission despite a drop in the INR-Value to <1.5 within 24 h after admission.

### 2.4. Definition of Major Bleeding

The occurrence and severity of peri- and postoperative bleeding < 24 h was judged by the surgeon, who performed the surgery according to the following definitions:

1. good hemostasis (for the type of surgery performed)
2. severe bleeding outside the surgical area with a drop in hemoglobin ≥ 2 g/dL or a need of >2 RBC transfusions within 24 h after admission
3. bleeding in the surgical area requiring re-surgery
4. bleeding in the surgical area that was unexpected and persistent and/or led to hemodynamic instability (drop in Hb value ≥ 2 g/dL or transfusion of >2 RBC) within 24 h after admission

The occurrence of severe bleeding > 24 h during the hospital stay (including clinically relevant non-major bleeding) was also documented.

### 2.5. Ethics

Approval of the study protocol was obtained from the institutional review boards of all participating centers. External independent monitoring ensured 100% site source data validity.

Due to the emergency nature of the conditions under investigation, patient information and informed consent should not interfere with or delay acute treatment. Therefore, with the approval of all ethics committees and institutional review boards, written informed consent was obtained from patients after the acute management phase. In the event of a patient’s inability to provide written informed consent, this was obtained from his/her legal representative. Data of patients who remained unconscious or died before a legal representative had been appointed were also included. This was explicitly approved by the ethical boards to prevent major bias caused by exclusion of the most severely affected patients [16]. The study complies with the Declaration of Helsinki. ClinicalTrials.gov (National Library of Medicine, 8600 Rockville Pike, Bethesda, MD 20894, USA) Identifier: NCT01722786.

### 2.6. Statistics

The statistical analysis focused on descriptive statistics (median, range, frequencies) and two-sided 95% confidence intervals. Primary analysis was the characterization of the primary endpoint (in-hospital mortality up to 30 days after admission) by a two-sided 95% confidence interval.

Data were given with median and 25–75% quartiles. Group comparisons between binary data were compared with the exact Fisher test and between ordinal or quantitative data with the Wilcoxon-Mann–Whitney test. Time to in-hospital mortality was compared with a log-rank test. All explorative tests used a significance level of alpha = 5%.

## 3. Results

### 3.1. Demographic Data of Included Patients

Overall, 78 patients were included, 44 were treated with a DOAC and 34 treated with VKA (phenprocoumon). In the DOAC-group, 52% (23/44) of patients were treated with apixaban, 32% (14/44) with rivaroxaban, 7% (3/44) with edoxaban und 9% (4/44) with dabigatran. Median age was 76 years. While nearly half of the DOAC-treated patients were women, only a quarter of the VKA-treated patients were female (*p* = 0.012). On admission, acute renal failure occurred to a similar extent in both groups (DOAC: 5% (2/44) vs. VKA: 9% (3/34); *p* > 0.20; see Table 1).

Indications for emergency surgery were mainly bone fractures (total: 35%, 27/78) or an acute abdomen (total: 30%, 23/78). Significantly more DOAC-treated patients suffered from bone fractures compared to VKA-treated patients (DOAC: 46%, 20/44 vs. VKA: 21%,7/34; *p*= 0.031). Numerically more VKA-treated patients needed open-heart surgery compared to the DOAC-treated patients (VKA: 21%, 7/34 vs. DOAC: 9%,4/44; *p* = 0.195).

Indication for oral anticoagulation was predominantly atrial fibrillation (total: 74%, 58/78) with a similar distribution in both groups (DOAC: 77%, 34/44; VKA: 71%, 24/34). The median CHADS-VASC Score was 5 in both groups (*p* > 0.20). The median bleeding risk score according to the modified HASBLED score (HASBLED score excluding the criterion “stability of INR”) was 2 in both groups. Concomitant treatment with antiplatelet or anti-inflammatory drugs was similar among both groups (antiplatelet drugs; DOAC: 14%, 6/44 vs. VKA: 15%, 5/34; *p* > 0.20; anti-inflammatory drugs; DOAC: 2%, 1/44 vs. VKA: 9%, 3/34; *p* > 0.20, see Table 1).

On admission, information on bleeding history was available for all VKA patients and for 43 of 44 DOAC patients. In total, 2 of 43 DOAC patients (5%) and 5 of 34 VKA patients (15%) had a history of bleeding (*p* = 0.23).

### 3.2. Management 

**Figure 1:**
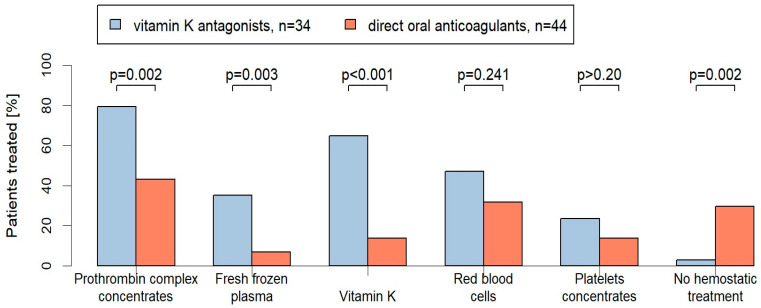
Proportion of hemostatic treatments [%] in the VKA- and DOAC-treated patients with emergency operations. Two-sided *p*-values are given for comparison.

Management was highly diverse (see Figure 1). While 80% of the VKA patients (27/34) received PCC, only 43% of the DOAC-patients (19/44) were treated with PCC (*p* = 0.002). FFP was also significantly more frequently applied in VKA-patients compared to DOAC-patients (35% (12/34) vs. 7% (3/44); *p* = 0.003). Vitamin K was less frequently applied in DOAC-treated patients compared to VKA-treated patients (14% (6/44) vs. 62% (21/34); *p* < 0.001). In total, 47% of the VKA patients (16/34) required RBC transfusions compared to 32% of the DOAC patients (14/44) (*p* = 0.241). Platelet concentrates were applied in both groups to a similar extend (DOAC: 14%, 6/44 vs. VKA: 24%, 8/34; *p* > 0.20). Moreover, 30% of the DOAC patients (13/44) received no hemostatic treatment compared to 3% (1/34) of the VKA patients (*p* = 0.002) (see Figure 1).

A delay of the procedures was observed to a similar extent in both groups (DOAC-group: 20%, 9/44; VKA-group: 21%, 7/34). In most cases, there was no delay (see Table 2).

#### Use of Specific Antidotes

Overall, four patients had been treated with dabigatran. Two patients needed trauma surgery, one was operated on because of an acute abdomen and one needed a CNS emergency surgery. Two of these patients were treated with idaruzicumab (5 g intravenously), one preoperatively and one postoperatively. In all four patients, the surgeons categorized the postoperative bleeding situation as good. Of note, andexanet alfa was not available in Germany at the time of recruitment.

### 3.3. Bleeding Complications

In 90% of the patients (70/78), hemostasis was categorized by the surgeon as “good” during the first 24 h peri- and postoperatively (93% of the DOAC-treated patients: 41/44; 85% of the VKA-treated patients: 29/34; *p* = 0.29).

In 7% of the DOAC patients (3/44) and in 21% of the VKA patients (7/34), major or clinically relevant non-major bleeding at the surgical site occurred until day 30 after admission (*p* = 0.09; see Table 3). 

Major bleeding complications did not differ depending on whether preoperative hemostatic treatment was applied or was not applied (7% (1/14) of the DOAC-patients vs. 19% (4/21) of the VKA-patients received preoperative hemostatic treatment compared to 7% (2/30) of the DOAC-patients vs. 24% (3/13) of the VKA-patients, who did not receive preoperative hemostatic treatment (*p* > 0.20)).

Most VKA-patients with bleeding complications (6/7) had an INR-level > 1.5 on admission but only three of these patients received PCC as a preoperative hemostatic treatment. An INR >1.5 was also observed in 24 of 26 non-bleeding VKA patients. The median INR on admission was comparable between VKA-patients without bleeding complications and VKA-patients with bleeding complications (INR 2.3 vs. INR 2.5, *p* = 0.923).

On admission, the median DOAC-concentration of the 3 DOAC-patients (3/44) with bleeding complication was numerically higher compared to the DOAC-treated patients without bleeding complications (41/44): 279 ng/mL vs. 104 ng/mL; *p* = 0.288. Preoperative PCC was only given to 1 of these patients with bleeding complications (see Table 3).

Postoperative hemostatic treatment until day 30 in patients with peri-surgery major bleeding was highly diverse. Most patients with bleeding complications received more than three different hemostatic treatments (see Table 3).

### 3.4. INR-Rebound

In 36% (12/33) of the VKA patients, an INR-Rebound was observed. Major bleeding occurred significantly more often in VKA patients with an INR-Rebound compared to those without an INR-Rebound (42% (5/12) vs. 5% (1/21); *p* = 0.016).

### 3.5. Thromboembolic Complications

None of the patients developed a venous or arterial thrombosis within 30 days after admission. 

### 3.6. In-Hospital Mortality (until Day 30 after Admission)

**Figure 2:**
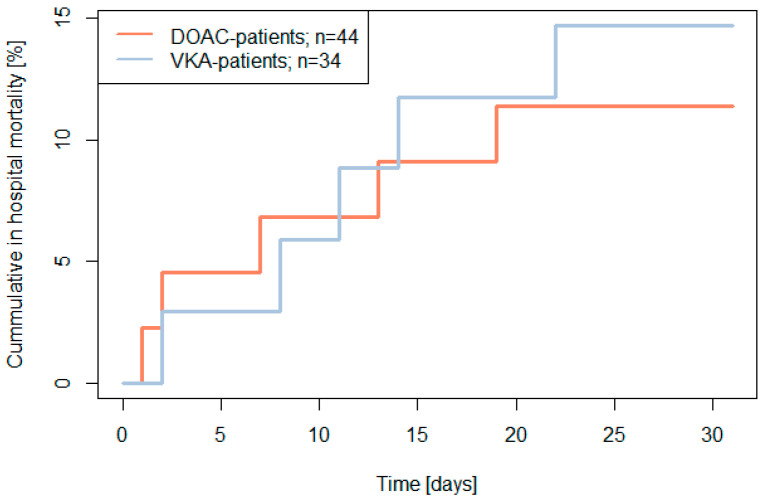
The 30-day in hospital mortality (primary endpoint) by baseline treatment in patients with emergency surgery (two-sided log rank test *p* > 0.20).

In-hospital mortality was 13% (10/78) with no significant difference between the two treatment groups (DOAC-patients: 11%, 5/44; VKA-patients: 15%, 5/34; *p* > 0.20) (see Figure 2). 

There was only one patient in the VKA-group who potentially died due to bleeding (3%; 1/34; total group: 1%, 1/78). Other reasons for 30-day in-hospital mortality were sepsis (DOAC: 7%, 3/44 vs. VKA 12%, 4/34; *p* > 0.20), acute heart failure (DOAC: 2%, 1/44 vs. 6%, 2/34; *p* > 0.20) and respiratory insufficiency (DOAC: 2%, 1/44 vs. 3%, 1/34; *p* > 0.20) without any significant differences between both groups.

## 4. Discussion

To our knowledge, the RADOA-registry is the first multicenter registry which prospectively analysed the use of hemostatic agents in either DOAC- or VKA-anticoagulated patients requiring emergency surgery within 24 h after hospital admission. Our elderly patient population (median age: 76) was mainly anticoagulated because of atrial fibrillation. Most frequent indications for emergency surgery were bone fractures and acute abdomen.

Similar demographic data was presented in a recently published French registry, the GIHP-NACO-registry, which analyzed the management of urgent invasive procedures in patients treated with DOAC [17]. This registry included 478 DOAC-treated patients with a median age of 79, who needed an urgent invasive procedure within 48 h after admission to hospital.

Compared to our patient population, the French registry included a study population with a lower bleeding risk since 34% of their patients only needed a minor procedure such as pleural punctures, skin and organ biopsies, or tooth extraction. Moreover, 50% of the invasive procedures were classified as “expedited” which required treatment within days instead of hours. Although many of the procedures (43%) were delayed, excessive bleeding was observed in 13% of all procedures and hemostatic agents were only administered in 16% of the procedures.

This is in marked contrast to our registry, in which only 20% of the affected DOAC patients had their surgery postponed and 70% of DOAC-treated patients received hemostatic treatment. Although our DOAC-patients had more major surgeries compared to the French registry, only 7% of the RADOA-patients experienced major bleeding, potentially because of more aggressive prohemostatic treatment.

The difference in clinical severity between the two registries is also reflected by the 30-day in-hospital mortality rate. It was significantly lower in the French registry (5.9%) than in our registry (13%). Mortality in our registry was more comparable with the mortality rate in the REVERSE-AD study (13%) which investigated the effects of the specific antidote idarucizumab in dabigatran-treated patients needing emergency surgery (n = 202) [10]. Moreover, a similar 30-day mortality of 15% was presented in a small subgroup population (n = 21) of a Dutch cohort study that prospectively analyzed the management and outcome of DOAC-treated patients who needed urgent interventions within 8 h after admission to hospital [18].

In contrast to the French registry and the Dutch study, our registry also included the management of VKA-treated patients needing emergency surgery. Our results show that the hemostatic treatment was highly diverse with significant differences between the VKA- and the DOAC-groups. Not surprising, VKA-patients received PCC almost twice as often as the DOAC-patients. Also, FFP and Vitamin K was significantly more frequently applied in VKA-patients compared to DOAC-patients. In addition, 30% of the DOAC-treated patients did not receive any hemostatic treatment compared to only 3% of the VKA-treated patients (*p* = 0.002).

More patients in the VKA group had a major or clinically relevant non-major bleeding at the surgical site (VKA: 21% (7/34) vs. DOAC: 7% (3/44); *p* = 0.09) which was often triggered by an INR-rebound, a phenomenon which is frequently observed in phenprocoumon-treated patients due to its long half-life [19]. Only 3 of the 7 VKA patients with major bleeding peri- and/or postoperatively received PCC before the emergency surgery, despite 6 out of 7 having INR values of >1.5 before surgery.

### Limitations

The RADOA registry is subject to all limitations that are typical for observational studies, including patient selection bias, subjective and non-standardized treatment decisions or reporting bias. In clinical routine, such registry data can also be influenced by variations in surgery indications or surgeons experience in performing the emergency procedure or evaluating the bleeding situation. The bleeding risks of different emergency surgeries are different, and an uneven distribution of different interventions may lead to different bleeding risks in the respective patient population.

Moreover, the small number of included patients limits the transferability of the results to the corresponding patient population. Randomized, controlled and prospective studies with higher numbers of study population are needed to verify our results.

## 5. Conclusions

Despite heterogeneous management approaches, 93% of the DOAC-treated patients and 85% of the VKA-treated patients showed good bleeding control during and after emergency surgery within 24 h after admission. A delay of the procedure was needed in only 20% of the patients. The 30-day in-hospital mortality was comparable in both patient groups. DOAC-treated patients required less prohemostatic treatment than VKA patients.

## Figures and Tables

**Table 1:** Characteristics of patients at baseline.

	Total (n = 78)	DOAC ^4^ (n = 44)	VKA (n = 34)	*p*-Values ^1^
Demographic data				
Female sex	31 (40%)	23 (52%)	8 (26%)	0.012
Age (years)	76.5 (43–94)	75.5 (43–94)	76.5 (46–91)	>0.20
Acute renal failure	5 (6%)	2 (5%)	3 (9%)	>0.20
Surgery type				
traumatic	31 (40%)	24 (55%)	7 (21%)	0.003
fractures	27 (35%)	20 (46%)	7 (21%)	0.031
acute abdomen	23 (30%)	12 (27%)	11 (32%)	>0.20
GI tract	18 (23%)	9 (21%)	9 (27%)	>0.20
CNS	3 (4%)	2 (5%)	1 (3%)	>0.20
abscess	1 (1%)	1 (2%)	0 (0.0%)	--
Open-heart surgery	11 (14%)	4 (9%)	7 (21%)	0.195
vascular	7 (9%)	2 (5%)	5 (15%)	>0.20
other	6 (8%)	3 (7%)	3 (9%)	>0.20
Co-medication, indication for anticoagulation and bleeding risk				
Antiplatelet drugs	11 (14%)	6 (14%)	5 (15%)	>0.20
Anti-inflammatory drugs	4 (5%)	1 (2%)	3 (9%)	>0.20
Indication				0.155
atrial fibrillation	58 (74%)	34 (77%)	24 (71%)	
venous thrombosis	5 (6%)	4 (9%)	1 (3%)	
postoperative prophylaxis	4 (5%)	1 (2%)	3 (9%)	
arterial thrombosis	4 (5%)	1 (2%)	3 (9%)	
artificial heart valve	2 (3%)	0 (0%)	2 (6%)	
other	5 (6%)	4 (9%)	1 (3%)	
CHADS-VASC Score	5.0 (1.0–9.0)	5.0 (2.0–9.0)	5.0 (1.0–8.0)	>0.20
HASBLED Score ^2^	3.0 (0.0–5.0)	2.0 (0.0–4.0)	3.0 (0.0–5.0)	0.126
HASBLED Score modified ^3^	2.0 (0.0–5.0)	2.0 (0.0–4.0)	3.0 (0.0–5.0)	>0.20

^1^ Explorative *p*-values from two-sided tests (Fisher test, Wilcoxon-Mann-Whitney test) for comparison of the DOAC- vs. VKA-group without significance correction for multiple testing. ^2^ Modified HASBLED score (i.e., HASBLED score excluding the criterion “stability of INR”) is used in patients treated with DOAC while the HASBLED score including stability of INR is used in patients treated with VKA. ^3^ Modified HASBLED score (i.e., HASBLED score excluding the criterion “stability of INR”) is used in all patients. ^4^ Dosing data were available for 42 of 44 patients treated with DOACs on admission. 38% of these patients received a reduced DOAC-dose (16/42).

**Table 2:** Delay of procedures due to anticoagulation.

Delay of Emergency Surgery in Hours	DOAC (n = 44)	VKA (n = 34)
No delay	35 (80%)	27 (79%)
4–6 h	4 (9%)	4 (12%)
7–12 h	2 (5%)	1 (3%)
13–24 h	3 (7%)	2 (6%)

**Table 3:** Major and clinically relevant non-major bleedings in VKA- and DOAC-patients with emergency surgery.

Patient/ Anticoagulant	Age	Gender	INR/DOAC Level	Type of Surgery	Preoperative Treatment	Bleeding ≤24 h after Admission	Postoperative Hemostatic Treatment ≤24 h after Admission	INR-Rebound	Repeat Surgery	Bleeding > 24 h after Admission	Postoperative Hemostatic Treatment > 24 h after Admission	In-Hospital Death
VKA1	66	m	3.9	acute abdomen	PCC	yes	PCC, RBC, Vit K	yes	yes	no	-	no
VKA2	64	m	2.8	open heart surgery	TA	yes	PCC, FFP, TA, P, RBC, PC	no	no	no	-	no
VKA3	82	m	2.2	open heart surgery	no	yes	PCC, FFP, TA, P, RBC, PC	yes	no	no	-	yes, day 22 #
VKA4	53	m	1.4	open heart surgery	no	yes	PCC, FFP, TA, P, RBC, PC, F	no	no	no	-	no
VKA5	66	m	2.0	gluteal fasciitis	no	no	PCC	yes	yes	yes, day 3	PCC, FFP, TA, RBC, F, PCC, Vit K, aFVII, FXIII	yes, day 14 #
VKA6	81	m	3.1	subdural hematoma	PCC, TA	yes	PCC, RBC, Vit K	yes	yes	yes, day 2	PCC	yes, day 2 ##
VKA7	69	m	1.8	peripheral artery occlusion	PCC, Vit K	no	TA, P	yes	yes	yes, day 8	FFP, RBC, Vit K	no
DOAC 1 *	89	f	279 ng/mL	acute abdomen	PCC	yes	PCC, Vit k, TA	-	yes	yes, day 2	PCC	no
DOAC 2 **	74	m	333 ng/mL	open heart surgery	no	yes	-	-	yes	yes, day 1–3	PCC, FFP, RBC, PC, F, aFVII	no
DOAC 3 ***	72	m	62 ng/mL	open heart surgery	no	yes	PCC, Vit k, TA, P	-	no	yes, day 3–4	PCC, RBC, PC, F, Vit K	yes, day 7 #

VKA: Vitamin K antagonist; DOAC: direct oral anticoagulant; PCC: Prothrombin complex concentrate; FFP: Fresh frozen plasma; TA: Tranexamic acid; Vit K: Vitamin K; RBC: Red blood cells; PC: Platelet concentrate; P: Protamin; F: Fibrinogen concentrate; aFVII: Activated factor VII concentrate; FXIII: Factor XIII concentrate. * Apixaban2.5 mg twice daily; ** Rivaroxaban 20 mg once daily, *** Apixaban 5 mg twice daily. ^#^ septic shock, multi-organ failure; ^##^ fatal bleeding, septic shock, heart failure.

## Data Availability

Data are contained within the article.
